# Drug Therapy for Acute Bleeding From Portal Hypertensive Gastropathy

**DOI:** 10.1155/1995/70813

**Published:** 1995

**Authors:** Juan Carlos Lopez-Talavera, Roberto J. Groszmann

**Affiliations:** Digestive Diseases. V.A.M.C. Yale University School OfMedicine 950 Campbell Avenue West Haven CT 06516 USA


### DRUG THERAPY FOR ACUTE BLEEDING FROM PORTAL HYPERTENSIVE GASTROPATHY

### ABSTRACT

*Panes, J., Pique, J.M., Bordas, J.M., Llach, J., Bosch, J., Teres, J. and Rodes, J. (1994) Reduction of gastric hyperemia by glypress in and vasopressin administration in cirrhotic patients with portal hypertensive gastropathy, Hepatololgy; 19: 55–60.*

**Gastric mucosal perfusion is increased in portal-hypertensive gastropathy, and this may contribute to gastric bleeding from these lesions. Therefore drugs reducing gastric mucosal perfusion may be beneficial in the treatment of overt bleeding from portal-hypertensive gastropathy. In this study gastric mucosal perfusion was assessed in 28 cirrhotic patients with portal-hypertensive gastropathy under basal conditions and after double-blind intravenous administration of vasopressin (0.4 U/min), glypressin (2-mg injection) or placebo, with laser-Doppler flowmetry and reflectance spectrophotometry. Vasopressin and glypressin induced a significant increase in blood pressure and a decrease in heart rate. These effects were more pronounced in the vasopressin group. Both vasopressin and glypressin induced a sustained and similar reduction in gastric mucosal perfusion as assessed by laser-Doppler flowmetry (–36% ± 8% and –34% ± 6%, respectively; $p < 0.05$ with respect to basal values and with respect to the control group). Whereas placebo had no effect. Both drugs significantly reduced the oxygen content of the gastric mucosa; however, the impairment in mucosal oxygenation was greater ($p < 0.05$) in the vasopressin group (–17% ± 3%) than in the glypressin group (–6% ± 0.1%). We conclude that the increased gastric-perfusion in cirrhotic patients with portal-hypertensive gastropathy may be reduced by either vasopressin or glypressin. These findings support the use of these drugs in clinical trails treating bleeding portal-hypertensive gastropathy. The lower reduction in gastric mucosal oxygen content observed with glypressin could decrease the incidence of ischemic adverse events associated with the use of vasopressin. (HEPATOLOGY 1994; 19: 55–60.)**

KEYWORDS: Portal hypertension, portal hypertensive, gastropathy, glypressin, vasopressin.

### PAPER DISCUSSION

Bleeding from esophageal varices and/or portal hypertensive gastropathy (PHG) are common and lethal complications in patients with liver cirrhosis. In an attempt to treat these complications of portal hypertension, many investigators have been evaluating drugs which could decrease splanchnic blood flow and, thereby, reduce portal flow and pressure. In the present paper, the authors investigate the effects of vasopressin (VP) and glypressin (GLY) on gastric mucosal oxygenation and perfusion in cirrhotic patients with PHG by utilizing two endoscopic techniques, reflectance spectophotometry (RS) and laser-doppler flowmetry (LDF). The former gives an estimation of the oxygen and hemoglobin content of the gastric mucosa, and the latter estimates tissue perfusion. RS and LDF are the techniques most widely used during endoscopy to assess gastric mucosa perfusion. Although they are relatively accurate they may be influenced by other factors, including the number of red blood cell circulating in the gastric tissue and observer variability. Therefore, it is important to perform these studies under strict double blind conditions.

VP is a powerful vasoconstrictor,[1] which reduces blood flow to the entire splanchnic territory. By this mechanism, it can reduce portal pressure and hepato-portal and collateral blood flow. Nonetheless, VP is not without adverse clinical effects namely those derived from vasoconstriction of the systemic territory. Reduced coronary blood flow, cardiac output and heart rate and increased systemic vascular resistance are some of the hemodynamic changes induced by VP that may restrict its use in up to 25% of patients.[2] The association of VP and Nitroglycerin (NGC) enhances the reduction in portal pressure by reducing hepato and portal-collateral resistance while attenuating the systemic side effects of VP.[3] Therefore, if VP is used, it should always be associated with NGC.

Glypressin (GLY) is a synthetic VP analogue (triglycyl lysine vasopressin or terlipressin) that is slowly converted *in vivo* into VP by enzymatic cleavage of the triglycyl residues. This slow transformation in tissues allows the achievement of high local concentrations with low circulating levels (resulting in a lesser toxicity).[4] The study performed by Panes *et al.* demonstrates that cirrhotic patients treated with VP or GLY have a significant increase in arterial pressure and decrease in heart rate, gastric mucosal perfusion, gastric mucosal oxygen and hemoglobin content in comparison with placebo. In this study, using VP and GLY at the doses commonly used in the treatment of variceal bleeding, both drugs caused systemic hemodynamic changes, although these effects were less marked in the GLY group.

In the present study at the doses used, VP induced significantly more systemic hemodynamic effects demonstrated by a greater increase in arterial pressure and a larger reduction in heart rate. However, locally in the gastric mucosa, GLY seems to have a more marked vasoconstrictive effect. This finding suggests a specific effect of GLY in this regional bed. Surprisingly, this greater local vasoconstrictive effect of GLY is accompanied by a lesser reduction in gastric mucosa oxygenation than the one induced by VP. Perhaps, this is due to the detrimental systemic effects of VP which reduce Hb saturation. This finding is of practical interest because it suggests that GLY should be used in preference to VP for bleeding PHG.

Clinical trials show that GLY, in comparison to placebo treatment, has a higher efficacy in controlling bleeding and reducing mortality,[5,6] and when compared with VP, GLY shows significantly lower complications even when NGC is associated with VP.[7] It is important to note that in the study by Panes et al. the hemodynamic effects are observed promptly after GLY administration. This point lends support to the concept that GLY may have a direct vasoconstrictor effect by itself.

Somatostatin (SOM), another drug able to decrease portal pressure by selectively vasoconstricting the splanchnic circulation,[8] may act through the inhibition of splanchinic vasodilatory peptides. Because it has been shown that vasoconstriction and reduction of portal pressure is more marked after i.v. bolus injection rather than continuous infusion,[8] it is recommended that during the early hours of treatment, a bolus administration be given followed by a constant infusion. Actually, the authors of the present paper recently reported that the increased gastric perfusion that patients with liver cirrhosis and PHG demonstrate can be effectively decreased by SOM. The bolus injection caused a more pronounced reduction in gastric perfusion than a constant infusion did, suggesting a dose-related effect.[9] The greatest advantage of this drug seems to be the lack of side effects. Several trials comparing SOM with other active treatments show that SOM is highly effective in the treatment of variceal bleeding, and it is truly a safe therapy.

Octreotide (OCT) is a cyclic octapeptide analogue of SOM with the advantage of a longer biological half-life.[10] Reports differ regarding the effect the OCT has on reducing portal pressure. Although it produces a reduction in azygos, blood flow, this effect is no longer seen after one hour, despite continuous infusion.Pretreatment with OCT seems to ameliorate the increase in portal flow observed after postprandial hyperemia.[11] So far it is not clear, despite several reports, what the real efficacy of OCT is in controlling gastrointestinal bleeding secondary to portal hypertension. On this basis, studies with large number of patients, clear end-points and different schedules of treatment comparing OCT with other effective therapies are needed to assess definitive results.

In conclusion, by using endoscopic reflectance spectophotometry and laser-doppler flowmetry, Panes *et al.* demonstrate that both VP and GLY are effective in reducing the increased gastric perfusion observed in cirrhotic patients with PHG, providing evidence on applicability of these endoscropic techniques. To characterize the effect and theoretical advantages of GLY over VP, or over VP plus NGC, further studies are needed. Taking into account the theoretical advantage of less systemic hemodynamic repercussions, although there are already preliminary reports showing that GLY and SOM are equally effective in controlling bleeding,[12] it would be important to perform appropriate controlled trials comparing GLY, SOM and OCT.

### *References*

1. Altura, B. M., Altura, B. T. (1977) Vascular smooth muscle and neurohypophyseal hormones. *Fed. Proc.*, **36**, 1853–1860.
2. Conn, H. O., Ramsby, G. R., Storer, E. H., *et al.* (1975) Intraarterial vasopressin in the treatment of upper gastrointestinal hemorrhage: a prospective controlled clinical trial. *Gastroenterlogy*, **68**, 211–221.
3. Groszmann, R. J., Kravetz, D., Bosch J., *et al.* (1982) Nitroglicerin improves the hemodynamic response to vasopressin in portal hypertension. *Hepatology*, **2**, 757–762.
4. Blei, A. T. (1986) Vasopressin analogs in portal hypertension: different molecules but similar questions. *Hepatology*, **6**, 146–147.
5. Walker. S., Stiehl, A., Raedsch, R., Kommerell, B. (1986) Terlipressin in bleeding esophageal varices. A placebo controlled double-blinded study. *Hepatology*, **6**, 112–115.

6. Freeman, J. G., Cobden, M. D., Record, CO. (1989) Placebo-controlled trial of terlypressin (Glypressin) in the management of acute variceal bleeding. *J. Clin. Gastroenterol*, **11,** 58–60.
7. D'Amico, G., Traina. M., Vizzini, G, *et al.* (1994) Terlipressin or vasopressin plus transdermal nitroglycerin in a treatment strategy for digestive bleeding in cirrhosis. A randomized clinical trial. *J. Hepatol.*, **20,** 206–212.
8. Bosch, J., Kravetz, D., Rodes, J. (1981) Effects of somatostatin on hepatic and systemic hemodynamics in patients with cirrhosis of the liver: comparison with vasopressin. *Gastroentrology*, **80,** 518–525.
9. Panes, J., Pique, J. M., Bordas, J. M., Casadevall, M., Teres, J., Bosch, J., Rodes J. (1994) Effect of bolus injection and continuous infusion of somatostatin on gastric perfusion in cirrhotic patients with portal-hypertensive gastropathy. *Hepatology*, **20,** 336–341.
10. Ericksson, L. S., Brundin T., Soderlund, C., Wahren, J. (1987) Hemodynamic effects of long-acting somatostatin analogue in patients with liver cirrhosis. *Scand. J. Gastroenterol*, **22,** 919–925.
11. Buonoamico, P., Sabba, C., Garcia-Tsao, G., Berardi, E., Antonica, G., Ferraioli, G., Jensen, J. E., Lerner, E., Taylor, K. J. W., Albano, O., Groszmann, R. J. Octreotide blunts post-prandial hyperemia in cirrhotic patients: a double-blind randomized echo-doppler study. *Hepatology* (in press).
12. Variceal Bleeding Study Group. Spain. (1994) Glypressin vs somatostatin in the treatment of acute variceal bleeding in patients with cirrhosis. A double-blind randomized controlled trial. *Hepatology*, **19,** 1361 (Abstract).

Juan Carlos Lopez-Talavera, M.D.
Roberto J. Groszmann, M.D.
Digestive Diseases. V.A.M.C.
Yale University School Of Medicine
950 Campbell Avenue. West Haven, 06516 CT. U.S.A.

